# Few Layered Oxidized h-BN as Nanofiller of Cellulose-Based Paper with Superior Antibacterial Response and Enhanced Mechanical/Thermal Performance

**DOI:** 10.3390/ijms21155396

**Published:** 2020-07-29

**Authors:** M. Onyszko, A. Markowska-Szczupak, R. Rakoczy, O. Paszkiewicz, J. Janusz, A. Gorgon-Kuza, K. Wenelska, E. Mijowska

**Affiliations:** 1 Faculty of Chemical Technology and Engineering, Department of Nanomaterials Physicochemistry, West Pomeranian University of Technology, Szczecin, Piastow Ave. 42, 71-065 Szczecin, Poland; Ewa.Borowiak-Palen@zut.edu.pl; 2 Faculty of Chemical Technology and Engineering, Department of Chemical and Process Engineering, West Pomeranian University of Technology, Szczecin, Piastow Ave. 42, 71-065 Szczecin, Poland; Agata.Markowska@zut.edu.pl (A.M.-S.); Rafal.Rakoczy@zut.edu.pl (R.R.); Oliwia.paszkiewicz@zut.edu.pl (O.P.); 3 Arctic Paper Kostrzyn SA, ul. Fabryczna 1, 66-470 Kostrzyn nad Odra, Poland; joanna.janusz@arcticpaper.com (J.J.); aleksandra.gorgon@arcticpaper.com (A.G.-K.)

**Keywords:** nanoparticles, cellulose fibers, hexagonal boron nitride, antibacterial activity

## Abstract

In this study, hexagonal boron nitride nanosheets enriched with hydroxyl groups (h-BN-OH) were successfully grafted on the surface of cellulose fibers after the simple and effective exfoliation and oxidation of bulk h-BN. OH groups of h-BN-OH and the ones presented on the surface of cellulose fibers interacted via hydrogen bonding. Both spectroscopic (FT-IR, XRD) and microscopic (TEM, SEM, and atomic force microscopy (AFM)) methods results proved the successful functionalization of the cellulose fibers with the nanomaterial. Modified cellulose fibers were used to prepare paper sheets samples with different concentrations of the nanomaterial (1 wt %, 2 wt %, and 3 wt %). All the samples were tested for the antibacterial properties via the colony forming unit method and exhibited good performance against both Gram-negative (*E. coli)* and Gram-positive (*S. epidermidis*) model bacteria. Additionally, the influence of the volume of working bacterial suspension on the antibacterial efficiency of the obtained materials was examined. The results showed significantly better antibacterial performance when the volume of bacterial suspension was reduced. Mechanical properties of the paper samples with and without nanofiller were also characterized. Tensile strength, tearing strength, and bursting strength of the paper samples containing only 2 wt % of the nanofiller were improved by 60%, 61%, and 118% in comparison to the control paper samples, respectively. Furthermore, the nanofiller improved the thermal properties of the composite paper—the heat release rate decreased by up to 11.6%. Therefore, the composite paper can be further explored in a wide range of antibacterial materials, such as packaging or paper coatings

## 1. Introduction

Cellulose is one of the most abundant biopolymer on the earth: it is the main constituent of plant cell walls and is also synthesized by algae, fungi, and some bacteria [1,2]. It represents about 1.5 × 10^12^ tons of annual biomass production. In this case, it can be considered as an inexhaustible organic material [3]. This durable, biodegradable, and renewable biopolymer has been extensively used by the society for thousands of years in its native form, predominantly in paper and textile products, as well as in cosmetics and pharmaceutics. Nowadays, due to the high demand for products made of sustainable, renewable, nontoxic, and non-petroleum based resources, the natural polymer industry is undergoing rapid growth. This is due to the increase the in negative effects of synthetic materials on the environment and health of living organisms, as well as the depletion of fossil resources. As it is crucial for the next generation and for our planet well-being to substitute non-biodegradable materials with those that are eco-friendly, more and more researches have recently been focusing on cellulose functionalization [4].

This fascinating biopolymer contains three hydroxyl groups in each d-glucose unit which provide a large number of modification possibilities and enable to obtain materials with various features that can be further used in a range of applications. Such material plays an important role in safety and quality products; thus, certain requirements should be met. Depending on the field, good mechanical properties, antibacterial activity, moisture and gas barrier properties, or tensile strength are the most important qualities for cellulose materials. Among them, antibacterial paper holds a great promise for numerous applications, e.g., food wrapping, sanitary and hospital paper, and medical appliances packaging. Several organic and inorganic components with antimicrobial functions have been used to impart the antimicrobial properties into the native cellulose fibers and improve performance of biobased materials. Nanoparticles are one of the groups of compounds exploited in functionalization of polymers due to the beneficial properties such as high surface area, good reactivity, facile functionalization and modification, as well as high aspect ratio [5]. To date gold [6] and silver nanoparticles [7], titanium oxide [8], and CuO or ZnO [9] have been well characterized and utilized as cellulose modifiers. However, lately, some reports have occurred proving that specific metals, e.g., silver, can be accumulated in the organs of living organisms [10]. Graphene and its derivatives are another group of compounds that have been used to modify cellulose fibers. On the one hand, these organics exhibit great antibacterial potential [11] and ability to improve physical properties while incorporated into polymer matrix. On the other hand, they exhibit some drawbacks that limit their application in the paper industry, e.g., high cost of bulk production, and dark color which decreases the esthetic values of cellulose materials. Thus, it is necessary to search for new economically viable, environmentally friendly, and easily synthesized antibacterial materials.

Hexagonal boron nitride, which is considered as a structural analogue of graphene [12], has received an enormous amount of attention in the last few years [13,14], due to its versatile features: excellent thermal and mechanical properties, chemical stability [15], low density [16], and biocompatibility [17], to name few. The latter along with unique mechanical strength allow its application in composite materials [18] to enhance their properties, especially for the paper-making industry. In comparison to graphene, this two-dimensional layered nanomaterial possesses several advantages. First of all it can be produced on a large scale with lower costs than graphene or its derivatives. It has high thermal stability and good mechanical properties comparable to graphene [19]. However it is white-colored, thus it is more preferable as filler in the packaging industry. It is understandable to think that its antibacterial potential could be similar to that one of graphene, considering their structural analogy and the resemblance of some features. Despite all these facts, until now, there have been only a few papers on the investigation of boron nitride antimicrobial properties [12], as well as cellulose and boron nitride composites [20]. One of the h-BN disadvantages to be even wider explored is lack of water solubility. Therefore, we propose a facile route to exfoliate and induce the hydrophilic functional groups on their surface, making it hydrophilic and chemically active with cellulose fibers structures.

**Figure 1:**
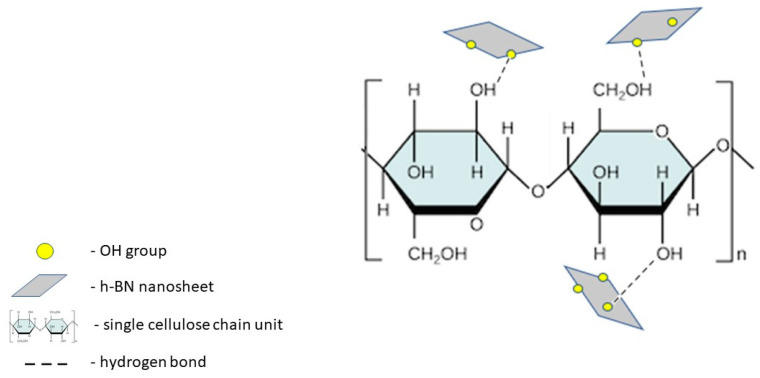
Schematic illustration of the interaction between cellulose and h-BN-OH via hydrogen bonding.

In our experiments, we successfully exfoliated hexagonal boron nitride from bulk sample with the assistance of ultrasounds. Its ultrathin, few-layered structure was confirmed via atomic force microscopy (AFM) analysis. Modified/simplified Hummer’s method was employed to produce h-BN nanosheets enriched with hydroxyl groups (h-BN-OH) [21]. Therefore, the interaction via hydrogen bonding between OH groups in h-BN-OH and the ones presented on the surface of cellulose fibers was revealed. Schematically, the interaction between the composite components is depicted in Figure 1.

The yield of the functionalization process as well as the morphology of materials were examined with spectroscopic (FT-IR, XRD) and microscopic (TEM, SEM) methods. As prepared, functionalized cellulose fibers were utilized to prepare paper sheet samples containing 1 wt %, 2 wt %, and 3 wt % of nanomaterial, respectively. All the samples were tested for their antibacterial activity and exhibited interesting properties against both Gram-negative and Gram-positive model bacteria. The influence of the volume of working bacterial suspension on the antibacterial performance of the obtained materials was also examined. Furthermore, the mechanical and thermal properties of the paper samples have been also described.

## 2. Results and Discussion

**Figure 2:**
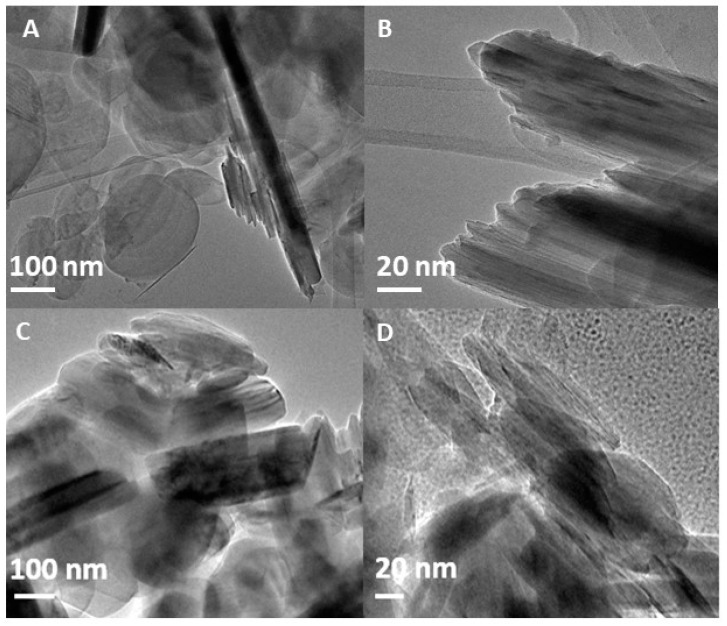
TEM images of h-BN (**A**,**B**) nanosheets and h-BN-OH (**C**,**D**) nanosheets with different magnifications.

Figure 2 presents TEM images of bulk h-BN (A-B) and h-BN-OH (C-D) obtained after its ultrasonication and acid mixture treatment. Due to the low thickness of h-BN, the samples are mostly translucent as the electron beam transmits through the material. However, it seems that sonication and acid treatment resulted in improved delamination of hexagonal boron nitride nanosheets, since more light passes through the h-BN-OH sample. In both cases flat, disc-shaped particles are observed indicating that the structure of the nanosheets remains ordered even after 12 h of ultrasonication. Moreover, the oxidation with acids did not affect the structure of boron nitride, as no pores or defects are detected on the surface of h-BN-OH.

**Figure 3:**
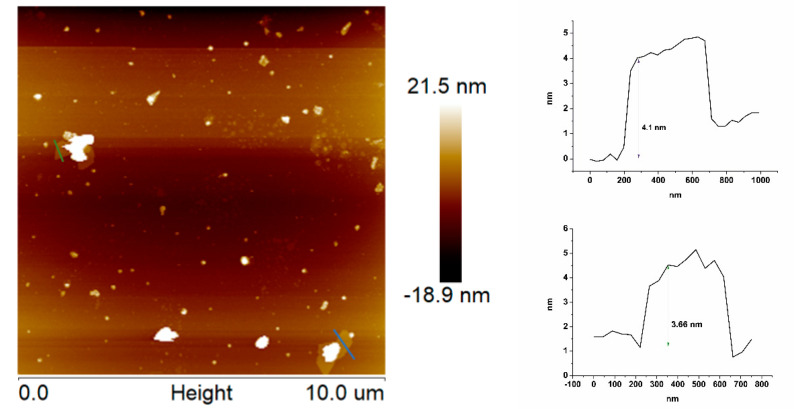
Atomic force microscopy (AFM) topography of h-BN nanosheets and the corresponding height profiles.

The thickness and lateral size of h-BN nanosheets after the exfoliation process were characterized by AFM, as shown in Figure 3. The sample for AFM measurement was prepared by deposition of a water suspension of h-BN on the silicon substrate, followed by drying at 90 °C for 2 h in an oven. The thickness of the observed h-BN particles was in the range of 3–5 nm, which corresponds to 10–15 layers of nanosheets. The lateral size of several particles was calculated and it revealed that the dimensions of individual nanosheets are quite similar, on average ~335 nm. The AFM analysis also indicated irregular and sharp edges of exfoliated h-BN nanosheets.

**Figure 4:**
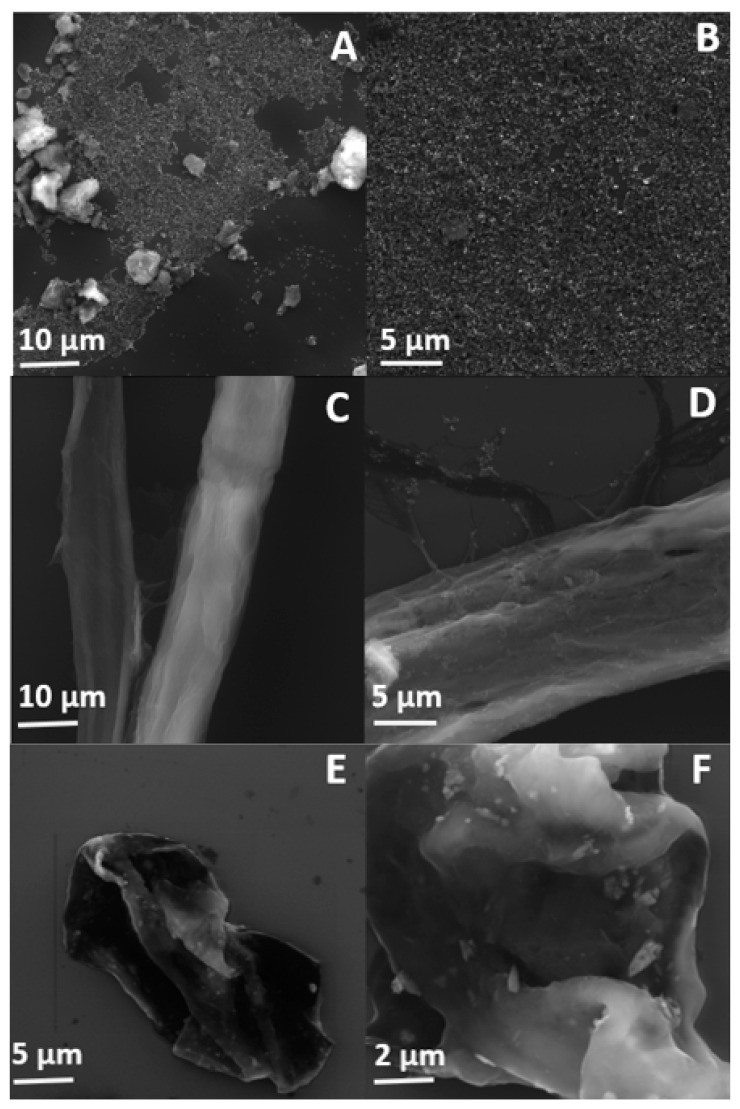
SEM micrographs of h-BN-OH nanosheets (**A**,**B**), cellulose fibers before (**C**) and after (**D**) the functionalization with h-BN-OH nanosheets, and cellulose fibers functionalized with h-BN-OH nanosheets after the ball-milling process (**E**,**F**).

The morphology of oxidized h-BN nanosheets as well as cellulose fibers before and after the functionalization process was characterized using scanning electron microscope. Figure 4A,B clearly shows that h-BN-OH nanosheets are mostly less than 1 μm in diameter, which is in a good agreement with AFM analysis. Although small amount of aggregates are detected in the SEM images, one can conclude that ultrasonication is a suitable and effective way to prepare exfoliated hexagonal boron nitride nanosheets from the bulk material.

SEM micrographs of the raw and functionalized cellulose fibers are presented in Figure 4C,D, respectively. The surface of the individual raw cellulose fibers is smooth and the cellulose macrofibrils run in the direction of the fiber. After the functionalization process, the presence of the distinct h-BN-OH nanoparticles is observed. The nanomaterial can be easily detected on the cellulose fibers. The nanoparticles are mostly uniformly distributed on the surface of cellulose fibers and only few aggregates are present, which implies that the mixing process can contribute to the separation of individual nanosheets.

In order to test the strength of the interaction of h-BN-OH with cellulose fibers, the sample was ball-milled for 30 min. Subsequently, an SEM analysis of the obtained sample was conducted. Figure 4E,F show that the fibers were destroyed during ball-milling, but the h-BN-OH are still present on the surface. The h-BN-OH nanosheets with the average size of less than 1 μm are clearly visible. The presence of the nanomaterial on the surface of cellulose fibers even after the milling process proves that interaction between h-BN-OH and cellulose is sufficiently strong for the industrial paper manufacturing in which high water pressure is involved.

**Figure 5:**
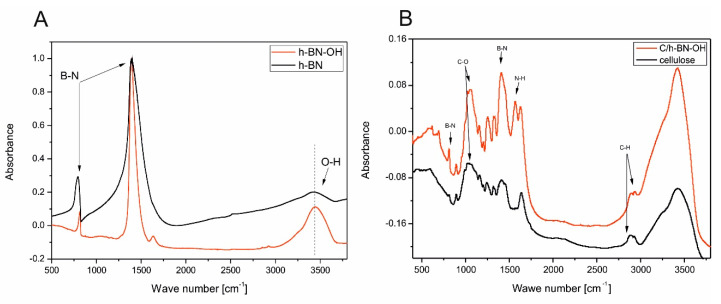
FT-IR spectra of: h-BN nanosheets (**A**) before (black curve) and after (red curve) the oxidation process and cellulose fibers (**B**) before (black curve) and after (red curve) the functionalization with h-BN-OH.

In order to characterize the structural differences between h-BN and h-BN-OH, the samples were subjected to FT-IR tests. As shown in Figure 5A, both materials exhibited a strong band at 1389 cm^−1^, which comes from the in-plane stretching vibration of BN and the absorption band at 790 cm^−1^ that corresponds to out-of-plane bending vibrations of BN [22,23]. The broad band near 3400 cm^−1^ is ascribed to the O-H stretching vibrations. In the case of h-BN, it can represent constitutional elements terminating the two-dimensional nanosheets [24] or the small number of OH groups on its surface [25]. It is clearly noticeable that this absorption peak is much more intense and sharp in the spectra of h-BN-OH, which indicates successful oxidation of raw material and enrichment h-BN nanosheets with OH groups. The additional weak band at 1365 cm^−1^ for h-BN-OH can be ascribed to adsorbed water molecules. It can be concluded that low temperature acid treatment of h-BN can be used to increase the amount of OH groups. Figure 5B presents FT-IR spectra of cellulose fibers from wood pulp before (black curve) and after (red curve) the modification with h-BN-OH. The characteristic absorption bands of cellulose are observed in two wave number regions: the fingerprint” region is between 400 and 1650 cm^−1^ and the O-H and C-H stretching vibrations are between 2700 and 3600 cm^−1^. The most intense peaks of pristine and functionalized cellulose fibers are observed at 3422 cm^−1^ and 3426 cm^−1^, respectively. These broad bands are characteristic for O-H stretching vibrations. They include inter- and intramolecular hydrogen bonds in cellulose and each distinct hydroxyl group gives a single stretching band. Thus, a mixture of inter- and intra-molecular hydrogen bonds is considered to cause the broadening of the OH band in the IR spectra. Another less intense, but noticeable peak around 2900 cm^−1^ is attributed to the stretching vibration of C-H bonds in polysaccharides [26,27,28]. Obvious changes can be seen in the FT-IR spectra functionalized cellulose compared with native cellulose in the range of 750–1400 cm^−1^. The observed additional bands are characteristic for h-BN-OH, which proves that it was successfully bonded with cellulose fibers.

**Figure 6:**
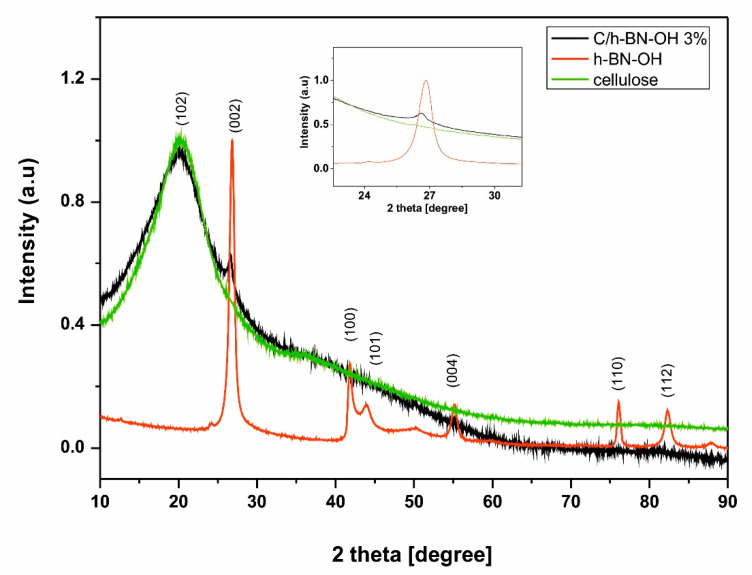
XRD patterns of h-BN-OH nanosheets (red curve) and cellulose fibers before (green curve) and after (black curve) the functionalization process.

X-ray diffraction was used to reveal the crystal structure of the obtained materials. Figure 6 represents XRD patterns of h-BN-OH nanosheets, raw cellulose fibers, and cellulose fibers functionalized with h-BN-OH. The XRD analysis of h-BN reveals the expected sharp and separated lines indicating high crystallinity of the sample. The principal reflection for h-BN-OH is located around 2θ ~ 26.8° and can be assigned to the (002) reflection of the graphite-like structure of the hexagonal boron nitride nanosheets [29]. The same signal can be observed in the XRD pattern of the functionalized cellulose sample, indicating the successful functionalization of the cellulose fibers. Additional and much weaker peaks characteristic for h-BN nanostructures are located around 2θ ~ 41.8°; 43.9°; 55.1°; 76.0°, and 82.3° [30]. However these signals cannot be seen in the pattern of functionalized cellulose, because they are screened by high intensity of the (102) crystallographic plane of cellulose [31].

**Figure 7:**
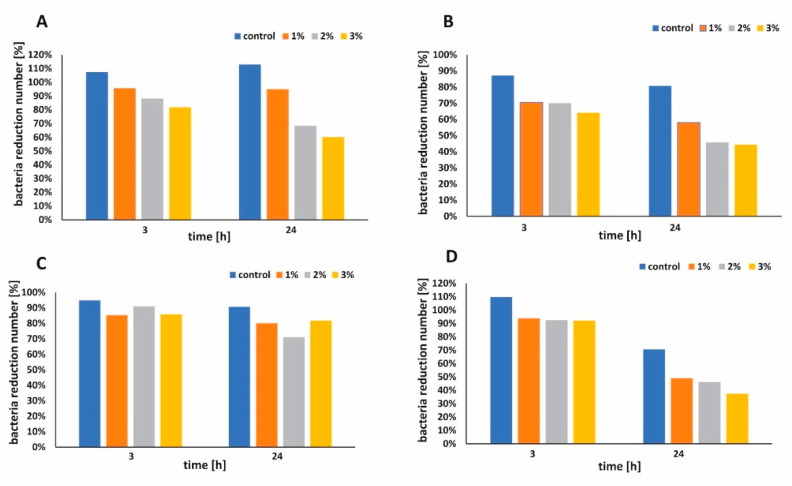
The antibacterial performance of functionalized 1, 2, and 3% h-BN-OH paper and control sample examined for varied bacterial suspension volumes: *E. coli* 275 mL (**A**), *E. coli* 55 mL (**B**), *S. epidermidis* 275 mL (**C**), and *S. epidermidis* 55 mL (**D**), after 3 and 24 h.

**Figure 8:**
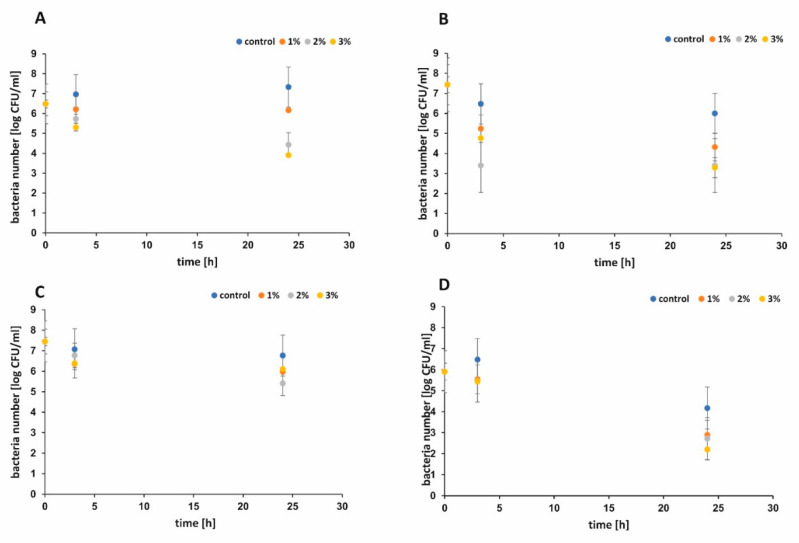
The bacterial survival rate [%] in contact with 1 wt %, 2 wt %, and 3 wt % h-BN-OH paper and control sample examined for varied bacterial suspension volumes: *E coli* 275 mL (**A**), *E coli* 55 mL (**B**), *S. epidermidis* 275 mL (**C**), and *S. epidermidis* 55 mL (**D**), after 3 and 24 h.

The antibacterial performance of functionalized paper and control sample against *Escherichia. coli and Staphyloccus epidermidis* were shown in Figure 7A–D, respectively. During the first 3 h, functionalized paper did not show significant antibacterial activity when the larger volume of bacterial suspension was used (Figure 7C). When the smaller volume of bacterial suspension was applied, all functionalized papers presented better antimicrobial activity against *E. coli* (Figure 7B,D). The bacterial survival rate [%] was examined and the results are presented in Figure 8A,B for *E. coli* and in Figure 8C,D for *S. epidermidis,* respectively. Apparently, the raw cellulose paper (control) hardly showed an inhibitory effect, whereas 1 wt %, 2 wt %, and 3 wt % h-BN-OH modified papers exhibited significant antibacterial activity. The inhibition ability against Gram-positive *S. epidermidis* was better in comparison to Gram-negative *E. coli* independently on the experimental series (Figure 8A–D). Additionally, in the case of *E. coli*, the antibacterial activity of paper functionalized with h-BN-OH was concentration-dependent. When the concentration of nanomaterial increased (from 1 wt % to 3 wt %), the linear reduction of bacteria population was observed independently on experimental series (Figure 8A,B). In the first series of experiments, the supplementation with 3 wt % of h-BN-OH caused inactivation of almost 19.3% (survival rate = 81.7%) of bacteria after 3 h of contact time and almost 40% (survival rate = 60.2%) of bacteria within 24 h of contact time. In the second series of experiments, the inactivation rate was almost 35.9% (survival rate = 64.1%) and 55.7% (survival rate = 44.3%) after 3 and 24 h, respectively. No correlation between the nanomaterial concentration and the *S. epidermidis* reduction was observed. Additionally, the results show that the antibacterial performance of functionalized cellulose samples against *S. epidermidis* was weaker in comparison to *E. coli*, when the volume of bacterial solution was larger (Figure 8A,C). The reason of faster inactivation of the Gram-negative bacteria might be due to the fact that its cell wall (composed of the peptidoglycan) is thinner than the one of the Gram-positive bacteria [32]. It may also indicate the mechanical mechanism of the antibacterial activity of h-BN-OH. The thick cell wall of *S. epidermidis* could protect the bacterial cell from being destroyed by sharp edges of h-BN-OH incorporated into the paper sheets. As a consequence of bacterial cell wall disruption, DNA damage is possible. A similar mechanism is observed for many carbon nanomaterials [33]. The physical (electrostatic) or chemical interaction between h-BN-OH and the Gram-negative outer membrane consisting of phospholipids, lipopolysaccharides (LPS), lipoproteins, and proteins are also possible [34]. Although BN materials do not exhibit toxicity and present rather good biocompatibility with human cells, it is noteworthy that an antibacterial effect was present at very low concentration of h-BN-OH.

To test the effect of the nanofiller on the mechanical properties of the paper, the control sample and the sample containing 2 wt % of h-BN-OH were prepared. The grammage of both paper sheets was approximately 70 g/m^2^. Table 1 summarizes the results of the examined mechanical properties. Tensile strength value of paper contains h-BN-OH increased by 60% over the non-functionalized paper. The same sample demonstrated 1.6 times greater value of tearing strength in comparison to the control paper. Moreover, the bursting strength value of the functionalized paper increased significantly—it was approximately 2.1 times greater than that of the control sample. One can conclude that as small amount of nanofiller as 2 wt % can significantly improve the mechanical performance of the paper.

**Figure 9:**
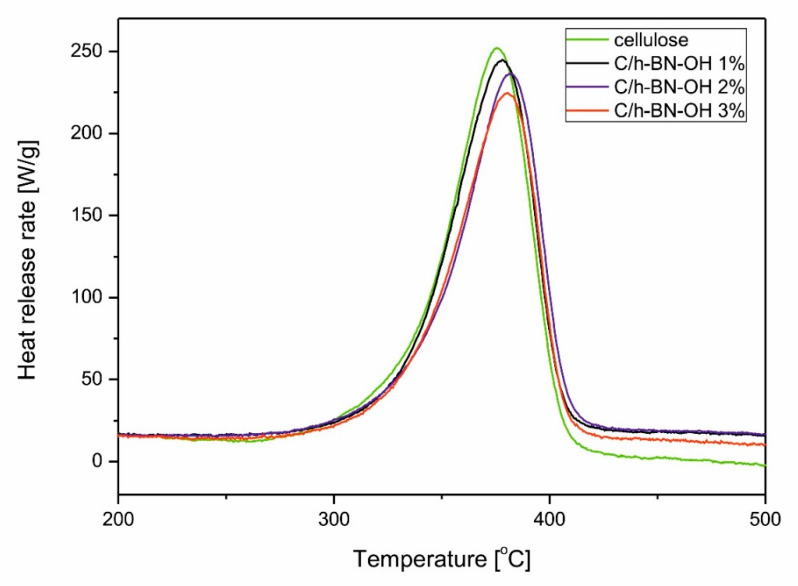
HRR curves of cellulose and cellulose/h-BN-OH with different concentration of nanofiller.

Since h-BN can be used as a flame retardant nonadditive for polymers [35,36], its thermal resistance properties have been also tested in composition with cellulose fibers. The typical heat release rate curves of the cellulose and cellulose/h-BN-OH paper sheets containing different loadings of nanofiller are presented in Figure 9 and the corresponding parameters are presented in Table 2. With the incorporation of h-BN-OH, the peak heat release rate significantly decreased. For example, when only 1% of h-BN-OH was added, the HRR decreased by 4.2% in comparison to pristine cellulose. Further addition of nanofiller resulted in more significant decrease in HRR up to 11.6% for the sample modified with 3% of h-BN. The presence of h-BN-OH nanosheets was also responsible for slight delay of thermal degradation and enhancement of thermal stability of treated cellulose fibers. Table 2 presents data that increased amount of nanofiller in cellulose elevates the temperature of maximum heat release rate. The results indicate that exfoliated and oxidized boron nitride nanosheets also boost flame retardancy of composite cellulose paper.

## 3. Experimental Procedures

### 3.1. Materials

Boron Nitride powder (~ 1 µm, 98%) was purchased from Sigma Aldrich (Poznan, Poland). Sulfuric acid (95%) and nitric acid (65%) were supplied from Chempur (Lodz, Poland). Bleached craft pulps consisted of short eucalyptus cellulose fibers and long wood cellulose fibers (4 gr/dm^3^) were received from Arctic Paper S.A. (Kostrzyn nad Odrą, Poland).

For the microbiological test nutrient broth, Brain-Heart Infusion, Standard Plate Count Agar and Brain-Heart Infusion Agar were purchased from BioMaxima S.A. (Lublin, Poland) As models, Gram-negative bacteria and model Gram-positive bacteria *Escherichia coli* K12 (ATCC 29425) and *Staphyloccocus epidermidis* (ATCC 49461) were used, respectively.

### 3.2. Preparation of h-BN Nanosheets

To obtain nanosheets of h-BN from bulk h-BN via microchemical cleavage, we used an ultrasonication method which is regarded as an effective approach to achieve few-layered nanosheets. First, 200 mg bulk of h-BN was placed in a flask and then 200 mL of isopropanol was added. The mixture was then placed in a glass vessel and the tip was immersed at half height. The mixture was sonicated for 12 h under continuous agitation at the frequency of 30 kHz. The obtained product was then washed with 500 mL of distilled water and dried in a drying oven at 100 °C.

### 3.3. Preparation of h-BN-OH

To produce oxidized h-BN nanosheets, the simplified Hummer’s method was applied. The process excluded the use of strong oxidation agent -KMnO_4_.

Briefly, 200 mg of dried exfoliated h-BN powder was placed in a round bottom flask under reflux and 13.5 mL of H_2_SO_4_ (95%) and 4.4 mL of HNO_3_ (65%) were carefully added. The dispersion was stirred for 30 min and then heated to 90 °C and stirred for another 12 h. After that, the product was cooled down, filtrated, and washed with distilled water until the pH value approached 7. The final product was dried in an oven at 90 °C for 12 h.

### 3.4. Fabrication of the Cellulose Paper Sheets Functionalized with h-BN-OH

To prepare functionalized cellulose paper sheet samples, 34 mL of cellulose pulp containing 1.36 g of short cellulose fibers and 3.5 mL of cellulose pulp containing 0.014 g of long cellulose fibers (9:1 *w*/*w*) were diluted in 400 mL of distilled water. The mixture was stirred with a magnetic stirrer at 60 °C for 20 min to obtain homogenous slurry. The desired amount of h-BN-OH corresponding to 1 wt %, 2 wt %, and 3 wt % of nanofiller per 1 g of dry cellulose fibers was added to the suspension and the mixture was stirred for 1 h, allowing h-BN-OH to bond to cellulose fibers via -OH bonds. The paper sheets were prepared using the filtration set [filtration set: all glass filter holder with NS and sintered disc-set]. Each sample was obtained from 18 mL of as-prepared functionalized cellulose pulp. The volume of the pulp was determined experimentally and allowed to produce smooth thin paper sheets weighted around 0.1 g with good reproducibility. The control samples were prepared in the same manner, but without the nanofiller. Before the antibacterial measurements, the samples were dried and stored at ambient conditions.

### 3.5. Characterization

The morphology of the obtained materials was examined via transmission electron microscopy (TEM, Tecnai F30), scanning electron microscopy (SEM, VEGA3 TESCAN) and atomic force microscopy (AFM, Nanoscope V MultiMode 8, Bruker). X-ray diffraction (XRD) patterns were carried out using X’Pert Philips Diffractometer with Cu lamp (Kα1 = 1.54056 Å) to investigate the crystal composition of the samples. Fourier transform infrared spectroscopy (FT-IR) was used to determine the functional groups that exist on the surface of cellulose fibers, h-BN and h-BN-OH. All the absorption spectra were recorded on Nicolet 6700 FT-IR Spectrometer

### 3.6. Antimicrobial Properties Characterization

The antimicrobial properties of functionalized paper and control sample were determined using Gram-negative bacteria *Escherichia coli* K12 (ATCC 29425) and Gram-positive bacteria *Staphyloccocus epidermidis* (ATCC 49461). The test microorganisms were cultivated in two types of liquid media–nutrient broth (NB) for *E. coli* and Brain-Heart Infusion (BHI) for *S. epidermidis* and incubated for 24 h at 37 °C. Then, the test culture was diluted using 0.85% sodium saline buffer to the final concentration 0.5 in *McFarland standard* to give a final concentration approx. 1 × 10^8^ CFU/mL. The experiments were carried out according to a previous report [37] with some modifications. For the antibacterial performance examination the samples of functionalized paper or raw cellulose paper (control) were cut into circles (Ø = 35 ± 1 mm; wt = 0.09 g ± 0.002 g) and transferred into 300 mL bottles containing 275 mL (first series) or 55 mL (second series) of working bacterial suspension, respectively. The capped bottles were placed in the incubator at 37 °C for 24 h. The suspension was continuously agitated using a magnetic stirrer (120 rpm). A volume of 1 mL of bacterial suspension was collected after 3 h and 24 h of incubation. Serial tenfold dilutions using 0.85% sodium saline solution were made and 0.25 mL were plated on appropriate solid media: Standard Plate Count Agar (PCA) for *E. coli* and Brain-Heart Infusion Agar (BHI) for *S. epidermidis*. The inculcated plates were incubated at a temperature of 37 °C for 24 h. Then, the visible colonies were counted and shown as log CFU/mL. The antimicrobial activity was expressed as bacterial survival rate after contact with the functionalized paper and control sample. All the experiments were repeated three times. Mean values ± and standard deviation (SD) were calculated using Microsoft Excel 2016.

### 3.7. Test of Mechanical Properties

We studied model paper samples manufactured under pilot conditions at the Arctic Paper S.A. Poland on a Rapid-Kothen 20 cm-wide paper making machine. The paper samples were made without the use of sizing and bleaching substances. Therefore, model paper samples were composed of pristine cellulose fiber compositions. All the mechanical tests were carried out at the Arctic Paper company in air-conditioned room (23 °C, 50% of the humidity level), according to ISO 5270 standard. The tensile strength measurements were conducted on the automatic tensile tester (model K465, Messmer Büchel, New Castle, DE, USA). Bursting strength was tested on the bursting strength tester (Messmer Büchel, New Castle, DE, USA). Tearing resistance was determined using Elmendorf apparatus (Lorentzen&Wettres, Kista, Sweden).

### 3.8. Tests of Flammability Properties

Micro Calorimeter (FAA MICRO Calorimeter) was used to investigate the thermal properties of control cellulose paper sheets and cellulose paper sheets functionalized with 1 wt %, 2 wt %, and 3 wt % of h-BN-OH, respectively. About 2.0 mg of the samples were heated in air atmosphere (80% of nitrogen and 20% of oxygen) at a constant heating rate of 1 °C/s from room temperature to 700 °C and the peak heat release rate (pHHR) was measured.

### 3.9. Statistical Evaluations

At least five tests on each specimen were performed for characterization of mechanical and flammability properties of the samples. The given results are the mean value with low ± standard deviation values.

## 4. Conclusions

In this work, the properties of cellulose paper sheets are improved due to the modification of cellulose fibers with h-BN-OH nanosheets. The incorporation of nanomaterial into the cellulose matrix and its successful bonding with fibers was proven by several microscopic and spectroscopic methods. Paper sheets prepared from modified cellulose fibers presented strong antibacterial performance against both Gram-negative (*E. coli)* and Gram-positive (*S. epidermidis*) bacteria in comparison to raw cellulose paper. Two experiments with different volume of bacterial suspensions were performed to examine the antibacterial properties. In each experiment linear concentration, the dependence of h-BN-OH on antimicrobial activity towards *E.coli* was observed. Moreover, better results were obtained against *E.coli* and *S. epidermidis* when the smaller volume of bacteria suspension was applied. After 24 h of contact with paper sheets modified with only 3% of h-BN-OH, the inactivation rate of *E.coli* was as high as 55.7% and 40.0% in smaller and higher volume of bacteria suspension, respectively. The incorporation of nanomaterial also influenced the mechanical and flame retardancy properties, which is an added value of the obtained antibacterial paper. Therefore, h-BN-OH loaded paper could be potentially used in various applications where antibacterial properties are required, in particular, in the packaging industry.

## Figures and Tables

**Table 1:** The effect of the nanofiller on the mechanical properties of paper.

	Control	h-BN-OH (2 wt %)
Tensile index [N m/g]	28.1 ± 1.1	45.1 ± 3.7
Tear index [mN m^2^/g]	4.6 ± 0.43	7.4 ± 0.64
Burst index [kPa m^2^/g]	1.42 ± 0.07	3.1 ± 0.08

**Table 2:** The effect of the nanofiller content on the HRR values and corresponding parameters.

Sample	h-BN-OH Content (% wt)	T_max_ (°C)	HRR (W/g)	Decrease in HRR (%)
cellulose	0	375.3 ± 13.37	242 ± 7.7	-
C/h-BN-OH 1%	1	377.8 ± 14.13	232 ± 6.4	4.2
C/h-BN-OH 2%	2	381.2 ± 12.72	223 ± 6.7	7.9
C/h-BN-OH 3%	3	381.5 ± 13.14	214 ± 5.6	11.6
